# Unveiling structural, chemical and magnetic interfacial peculiarities in *ε*-Fe_2_O_3_/GaN (0001) epitaxial films

**DOI:** 10.1038/s41598-018-25849-z

**Published:** 2018-06-07

**Authors:** Victor Ukleev, Sergey Suturin, Taro Nakajima, Taka-hisa Arima, Thomas Saerbeck, Takayasu Hanashima, Alla Sitnikova, Demid Kirilenko, Nikolai Yakovlev, Nikolai Sokolov

**Affiliations:** 1 RIKEN Center for Emergent Matter Science (CEMS), Wako, 351-0198 Japan; 2 Ioffe Institute, Saint-Petersburg, 194021 Russia; 3 Department of Advanced Materials Science, University of Tokyo, Kashiwa, 277-8561 Japan; 4 Institut Laue-Langevin, 71 Avenue des Martyrs, 38042 Grenoble, France; 5 Comprehensive Research Organization for Science and Society (CROSS), Tokai, Ibaraki, 319-1106 Japan; 6 Institute of Materials Research and Engineering, Agency for Science Technology and Research (A*STAR), 138634 Singapore, Singapore; 7 Present Address: Laboratory for Neutron Scattering and Imaging (LNS), Paul Scherrer Institute (PSI), CH-5232 Villigen, Switzerland

## Abstract

The metastable *ε*-Fe_2_O_3_ is known to be the most intriguing ferrimagnetic and multiferroic iron oxide phase exhibiting a bunch of exciting physical properties both below and above room temperature. The present paper unveils the structural and magnetic peculiarities of a few nm thick interface layer discovered in these films by a number of techniques. The polarized neutron reflectometry data suggests that the interface layer resembles GaFeO_3_ in composition and density and is magnetically softer than the rest of the *ε*-Fe_2_O_3_ film. While the in-depth density variation is in agreement with the transmission electron microscopy measurements, the layer-resolved magnetization profiles are qualitatively consistent with the unusual wasp-waist magnetization curves observed by superconducting quantum interference device magnetometry. Interestingly a noticeable Ga diffusion into the *ε*-Fe_2_O_3_ films has been detected by secondary ion mass spectroscopy providing a clue to the mechanisms guiding the nucleation of exotic metastable epsilon ferrite phase on GaN at high growth temperature and influencing the interfacial properties of the studied films.

## Introduction

The combination of magnetic and semiconductor materials within a single heterostructure provides vast opportunities for designing novel functional spintronic devices^1–3^. Among the known magnetic materials, iron oxides, though having a simple formula, exhibit rich variety of magnetic properties depending on the iron oxidation state and coordination. Metastable *ε*-Fe_2_O_3_, an exotic member of the iron oxides family, is the most intriguing phase not existing in the bulk and having been so far only crystallized in the form of nanoparticles^4–6^ and thin epitaxial films on a number of oxide substrates^7–9^. The *ε*-Fe_2_O_3_ phase is ferrimagnetic with largest magnetocrystalline anisotropy among the iron oxides responsible for coercivity values exceeding 2 T in the nanocrystalline form^4,10,11^ and 1 T in epitaxial layers^7^. Further, *ε*-Fe_2_O_3_ exhibits pyroelectric properties, room-temperature magnetoelectric coupling^12,13^ and non-linear magneto-optical effects^11,13,14^. Extending the successful attempts to stabilize epsilon ferrite on a number of oxide substrates, quite recently single crystalline layers of this material have been stabilized on GaN(0001) semiconductor surface by laser-assisted molecular beam epitaxy. The feasibility to grow the exotic *ε*-Fe_2_O_3_ phase along with three other iron oxide phases (Fe_3_O_4_, *α*-Fe_2_O_3_ and *γ*-Fe_2_O_3_) has been demonstrated^15^. The choice of the semiconducting material in this study is supported mainly by the wide usability of the gallium nitride in the modern optoelectronic and microelectronic devices such as bright LEDs, HEMT transistors for high-power, high-frequency, high-temperature and high radiation resistant applications^16,17^. Incorporating *ε*-Fe_2_O_3_ layer with controllable magnetization/polarization into a GaN-based device is supposed to provide additional functionality and therefore opens the way for the novel optoelectronic, electronic and spintronic applications^18,19^.

Comparing the results of the previously reported works on *ε*-Fe_2_O_3_ epitaxial films^7–9,12,20^ one can conclude that magnetic properties of the *ε*-Fe_2_O_3_ layer dramatically depend on the composition of the neighboring buffer layer and the chosen substrate. Interestingly, a peculiar double-hysteresis wasp-waist loop indicating the presence of an additional magnetically soft component was observed in *ε*-phase layers grown on SrTiO _3_ (111) (STO)^7,21^, AlFeO_3_ (AFO)/Nb:STO^12^, GaFeO_3_ (GFO)/Al_2_O_3_(0001)^8^, yttrium stabilized zirconia (YSZ)^9^ and GaN^15^ substrates and still has not clearly been understood. In contrast to this, ordinary rectangular or oval-shaped loops were demonstrated for *ε*-Fe_2_O_3_ thin films grown onto GaFeO_3_/STO(111) and GaFeO_3_/Al_2_O_3_(0001)^8,22^. Moreover, in the latter work Thai *et al*. reported that *ε*-Fe_2_O_3_ films got saturated in field of *B* = 0.5 T^8^, despite the saturation field of *ε*-phase being typically in the range of 2–3 T^4,10^. For *ε*-Fe_2_O_3_ nanoparticles, the step-like shape of the loop was attributed to the co-existence of a major *ε*-phase with a minor *γ*-Fe_2_O_3_ fraction^5,23^. Quite similarly, Corbellini *et al*. reported a ≈ 10 % fraction of magnetite (Fe_3_O_4_) in the PLD-grown Al-doped *ε*-Fe_2_O_3_ films resulting in non-zero magnetic moment at temperatures higher than 500 K^20^.

While the majority of the cited works report the need to grow a stand alone buffer layer of a *Pna*2_1_ space group material (GFO or AFO) prior to the *ε*-Fe_2_O_3_ film deposition, the ferrite-on-GaN technology is claimed not to require any buffer. In this paper, we show that a few nanometer-thick Ga rich transition layer with properties different from the main *ε*-Fe_2_O_3_ film is formed at the interface upon high temperature deposition. The transition layer is supposed to define the *Pna*2_1_ lattice structure of the further growing iron oxide layer thus leading to the nucleation of the exotic *ε*-Fe_2_O_3_ phase. In the present work we report the structural, chemical and magnetic peculiarities of the interface layer studied by a number of complementary methods that allow a separate evaluation of the structural and magnetic properties related to the bulk and interface film regions. In contrast to our previous report^15^ which focused either on the near surface region (reflection high-energy electron diffraction, atomic force microscopy, X-ray absorbtion spectroscopy) or on the bulk-integrated properties (X-ray diffraction, vibrating sample magnetometry), the present paper involves a depth resolved analysis of the *ε*-Fe_2_O_3_/GaN films performed by polarized neutron reflectometry (PNR), transmission electron microscopy (TEM) and secondary ion mass spectroscopy (SIMS).

## Experimental

The three micrometer-thick Ga terminated GaN (0001) layer acting as the host surface for the iron oxide deposition was fabricated by means of MOVPE on the commercial Al_2_O_3_ (0001) substrates. Epsilon ferrite films of 40–60 nm thickness were grown by means of laser molecular beam epitaxy from a stoichiometric Fe_2_O_3_ target ablated by pulsed KrF excimer laser radiation. The iron oxide deposition was performed in oxygen atmosphere at a pressure of 0.2 mbar using the growth conditions similar to those reported in our previous publication^15^. Despite using a lower growth temperature of 700 °C (800 °C in our previous report) we could confirm the epitaxial stabilization of *ε*-Fe_2_O_3_ phase by *in-situ* reflection high-energy electron diffraction (RHEED) reciprocal space mapping and *ex-situ* X-ray diffraction (XRD) methods. As was reported earlier the *ε*-Fe_2_O_3_ layer grows with the polar [001] axis oriented perpendicular to the substrate surface and the *ε*-Fe_2_O_3_ [100] easy magnetization axis parallel to one of the three equivalent GaN^1–10^ directions resulting in three crystallographic domains at 120° to each other.

**Figure 1:**
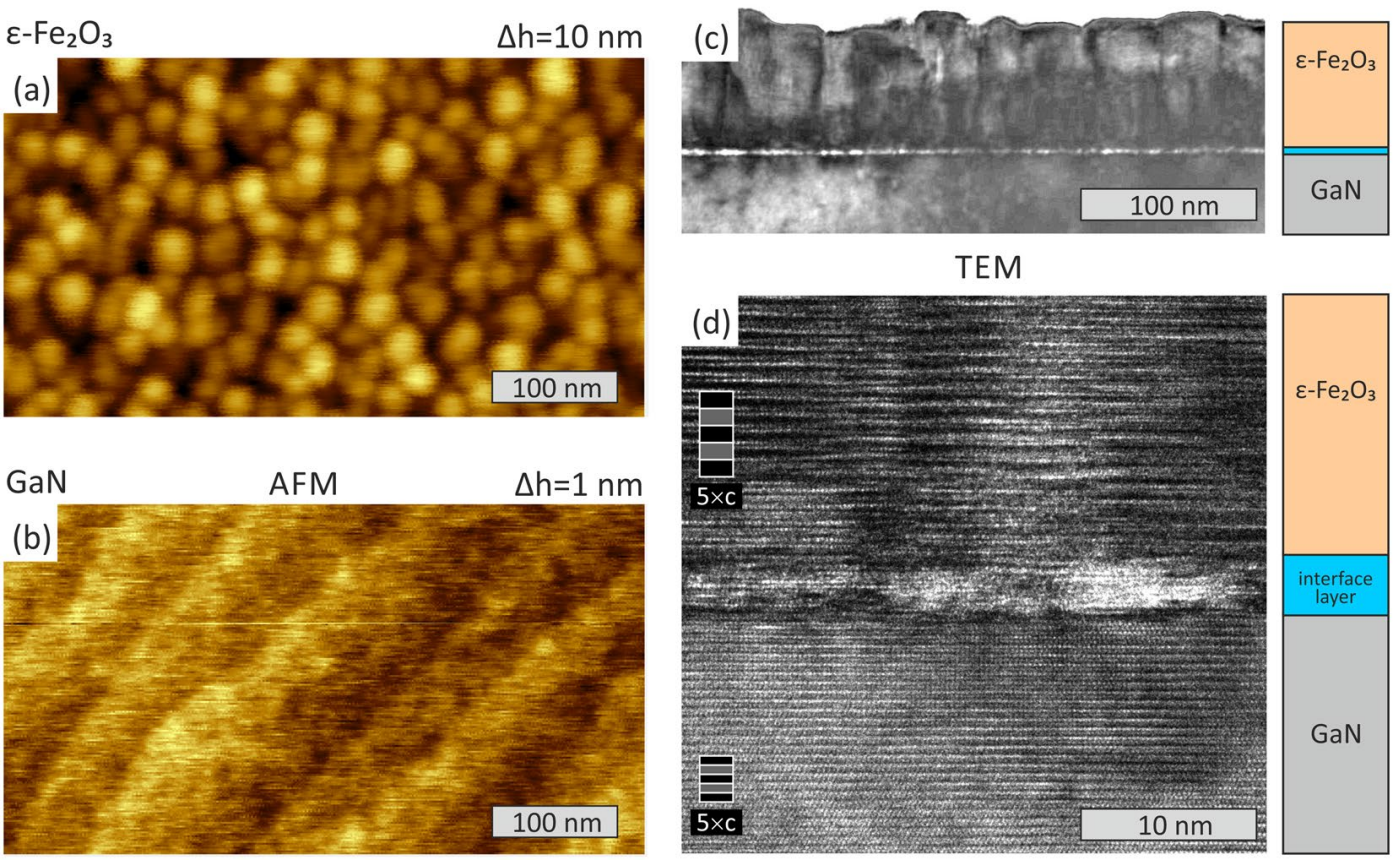
Atomic force microscopy images of the *ε*-Fe_2_O_3_ surface (**a**) and of the clean GaN surface (**b**). Cross-section transmission electron microscopy images of the 70 nm *ε*-Fe_2_O_3_ layer: the bright-field image (**c**) and high-resolution image (**d**).

As confirmed by atomic force microscopy, *ε*-Fe_2_O_3_ forms a uniform layer on the GaN step-and-terrace surface (Fig. 1a,b). On the nanometer scale the epsilon ferrite film surface shows mounds 25–35 nm in width and up to 10 nm in height. A more detailed investigation of the *ε*-Fe_2_O_3_/GaN morphology was carried out on a Jeol JEM-2100F transmission electron microscope (acceleration voltage 200 kV, point-to-point resolution 0.19 nm) operated in conventional bright-field and high-resolution imaging modes. TEM images presented in Fig. 1c,d confirm that the *ε*-Fe_2_O_3_ film is continuous with internal structure consisting of columns with the lateral size of 30–50 nm. The film surface roughness is a few nanometers peak-to-valley and caused by slightly different height of the neighboring columns. A similar structure with somewhat larger surface roughness was reported by other authors for *ε*-Fe_2_O_3_ layers grown on GaFeO_3_/STO, GaFeO_3_/YSZ surfaces^7–9,12^. The most likely reason for the columnar growth is the lower symmetry of the orthorhombic *ε*-Fe_2_O_3_ as compared to hexagonal GaN. At the early growth stage, individual islands have a threefold rotational degree of freedom in nucleation, leading to an in-plain rotation of 120° between grains. Apparently these defects persist throughout the island coalescence stage which results in the growth of vertical columns with antiphase boundaries in between them. The substrate interface of the epsilon ferrite film is much smoother than the surface. Interestingly, the high-resolution TEM suggests that a 4–5 nm thick interface layer with degraded crystalline quality is formed between GaN and *ε*-Fe_2_O_3_. This layer appears lighter in the TEM images which might indicate that this layer has a lower density. While the high resolution TEM image in Fig. 1 shows distinctly the atomic layers corresponding to c = 9.47 Å in *ε*-Fe_2_O_3_ and c = 5.189 Å in GaN, it is quite difficult to recognize a regular atomic structure in the transition layer at the interface. As was mentioned in our previous publication^15^ the RHEED patterns observed during growth of the first few nanometers are characteristic of a slightly disordered lattice with the lateral periodicity being reduced compared to that of *ε*-Fe_2_O_3_.

**Figure 2:**
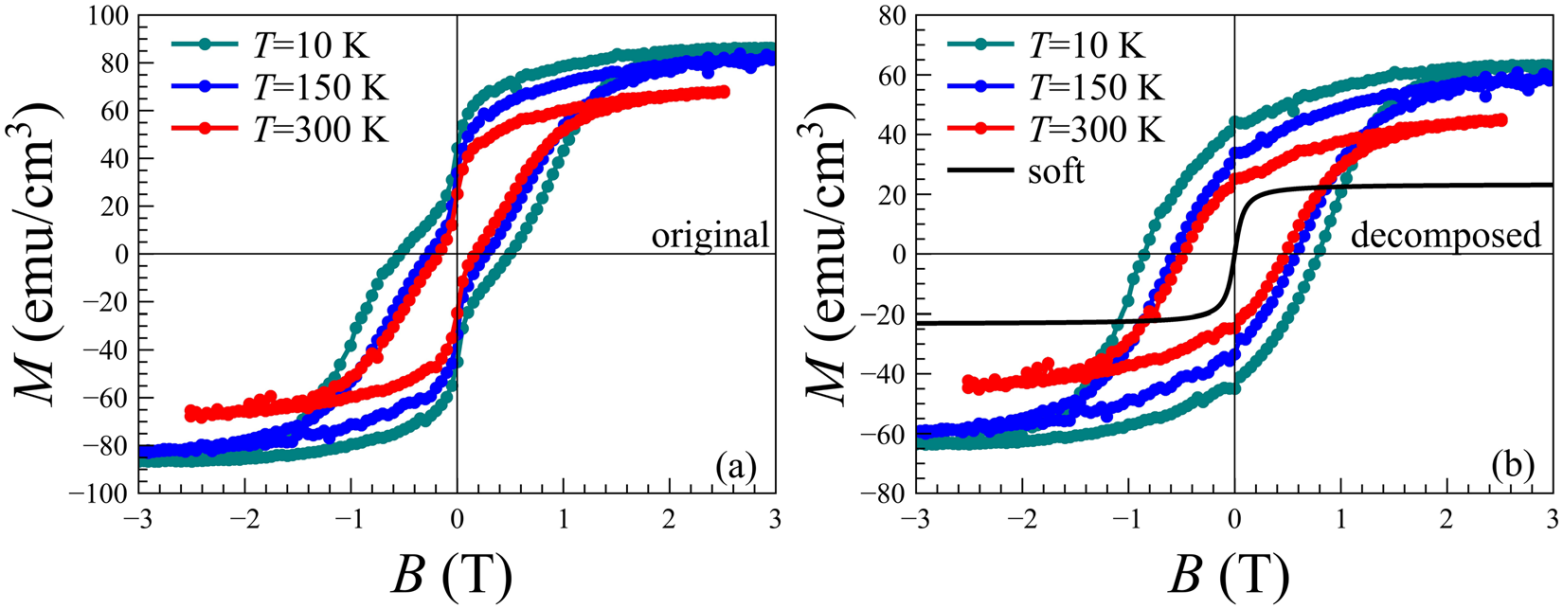
Magnetization loops of *ε*-Fe_2_O_3_ taken with the magnetic field applied in the sample plane after correction for the diamagnetic contribution from the GaN/Al_2_O_3_. Shown are original loops (**a**) and (**b**) decomposed into hard and soft magnetic components.

Magnetization measurements with magnetic field of up to 3 T applied in plane of the film were carried out using a Quantum Design 5XL magnetic property measurement system (MPMS) superconducting quantum interference device (SQUID) magnetometer in the temperature range from 10 to 380 K. Hysteresis loops measured in a 60 nm *ε*-Fe_2_O_3_ film at different temperatures are shown in Fig. 2a. Magnetic field was applied in the sample plane parallel to one of the [100] easy axis. For each temperature the loops were corrected for linear diamagnetic contribution of GaN/Al_2_O_3_ substrate. Saturation magnetization of about 80 emu/cm^3^ at *T* = 10 K is somewhat lower than the values reported for *ε*-Fe_2_O_3_ thin films and nanoparticles^4,12^. It is also lower than the values reported in our previous publication^15^ where *ε*-Fe_2_O_3_ layers were grown at a higher temperature of 800 °C. The coercive field *B*_*c*_ gradually increased as the temperature decreased from 180 mT at 380 K to 580 mT at 5 K.

The wasp-waist magnetization loop shown in Fig. 2a can be qualitatively decomposed to hard and soft component loops by subtracting 2*M*_*soft*_/*π*⋅arctan(*B*/*B*_*soft*_) function with temperature-independent *M*_*soft*_ = 25 emu/cm^3^ and *B*_*soft*_ = 76 mT, chosen to eliminate the magnetization jump at zero field (Fig. 2b). In this way the coercivity of the hard component can be estimated as 500 mT at RT and 800 mT at 10 K. These values are much closer to the characteristic *ε*-Fe_2_O_3_ coercivity but still lower than the 2 T values reported for *ε*-Fe_2_O_3_ nanoparticles^4,10^. Another reason for decreased coercivity is that when the applied field is parallel to the easy axis of one crystallographic domain, it makes 60° with the easy axes of the other two, making them switch magnetization in distinctly lower fields due to the presence of the off-easy-axis magnetic field component^24^. As it was noted by Ohkoshi *et al*.^13^, the coercivity dramatically depends on the nanoparticle size. Assuming that the *ε*-Fe_2_O_3_/GaN films consist of single domain columnar grains the *B*_*c*_ of 500 mT would correspond to the column size of 9–10 nm–much smaller than the size observed by TEM. It must be noted that the columns visible in the cross-section TEM images may have internal structure consisting of even smaller crystallographic domains. A more detailed investigation involving planar TEM studies is required to clarify the origin of the decreased coercivity.

Taking into account the fact that in thin films a phase separation often occurs at the interface^25–27^ we have conducted PNR measurements of the *ε*-Fe_2_O_3_/GaN heterostructures aimed at layer-resolved study of magnetization distribution inside the epsilon ferrite layer. The important technical aspects of our PNR experiment are described below, while the details of methodology can be found elsewhere^28,29^. Neutron reflectometry experiments were performed at the instruments D17^30^ (ILL, Grenoble, France) and BL17 SHARAKU^31^ (J-PARC MLF, Tokai, Japan) in polarized time-of-flight mode. The beamtime for room- and low-temperature PNR measurements was distributed between D17^32^ and SHARAKU instruments, respectively. Sample temperature and magnetic field at D17 instrument were controlled by 7 T vertical field cryomagnet with single-crystalline sapphire windows. Neutrons with wavelengths 4 < *λ* < 16 Å were used in the time-of-flight mode to maximize the incoming flux and keep the constant polarization efficiency in the whole *q*-range. Polarization of the direct beam *P*_0_ = 99% was measured by an analyzer. The minimum guide field required to prevent beam depolarization was *B*_*g*_ = 50 mT, likely due to a residual remanent field in the high-field superconducting magnet coils. The experiment was performed without polarization analysis. *R*^+^ and *R*^*−*^ reflectivities were acquired without distinction of the spin-flip and non spin-flip components. Intensity of the reflected beam was detected by two-dimensional ^3^He position-sensitive detector (PSD) and corrected for polarization efficiency, reflected beam divergence and wavelength resolution^33,34^. At SHARAKU the magnetic field was applied parallel to the incident neutron beam and spin polarization by horizontal-field 4 T cryomagnet. Neutron beam with the wavelengths 2 Å < *λ* < 8 Å was provided with constant polarization efficiency of *P*_0_ = 98.5%. *R*^+^ and *R*^−^ reflectivities were detected by a ^3^He gas tube PSD.

**Figure 3:**
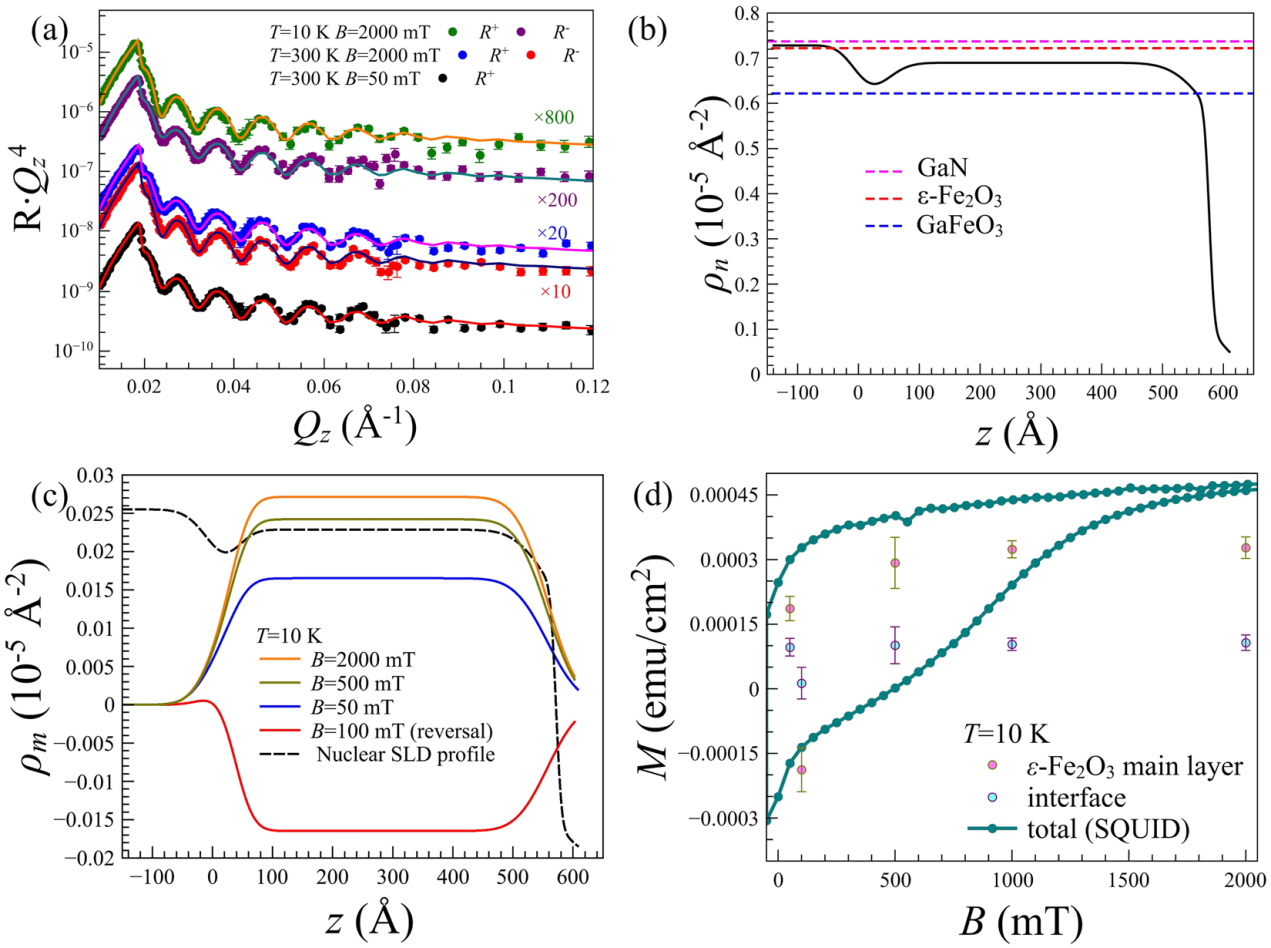
PNR curves measured at (**a**) *T* = 300 K (*B* = 50 and 2000 mT) and at *T* = 10 K (*B* = 2000 mT). Symbols represents the experimental data while the solid lines are calculated. The curves are shifted vertically for clarity. (**b**) Nuclear SLD profiles delivered by fitting routine. (**c**) Magnetic SLD profiles obtained at *T* = 10 K and different magnetic fields. Dashed line is corresponding to the *ρ*_*n*_ in the arbitrary units. (**d**) Layer-specific and total area-normalized magnetization curves for *ε*-Fe_2_O_3_ film at *T* = 10 K.

PNR curves *R*(*Q*_*z*_) measured at temperatures *T* = 300 K and *T* = 10 K shown in Fig. 3a are multiplied by ${Q}_{z}^{4}$ to compensate the Fresnel decay and provide better visibility to all the features of the reflectivity function. Note, that all the curves are shifted along vertical axis for clarity reasons: the multiplication factor is indicated for each curve. The splitting of *R*^+^(*Q*_*z*_) and *R*^−^(*Q*_*z*_) is proportional to the net magnetization of the film along the field direction. Because of the relatively small magnetic moment of the *ε*-Fe_2_O_3_ film, the splitting is hardly distinguishable at *T* = 300 K, but clearly observed after the sample was cooled to *T* = 10 K in magnetic field of *B* = 2 T. A set of PNR measurements was performed on the field-decreasing hysteresis loop branch from +62 T to +50 mT where both hard and soft magnetic components are magnetized positive (Fig. 1 in Supplemental Information). After that a negative field of *B* = −2 T has been applied to achieve negative saturation and another measurement was carried out at *B* = +100 mT. This point on the field-increasing loop branch (Fig. 2) corresponds to the state in which the soft magnetic component has already switched to the positive saturation while the hard magnetic component is still in negative remanence.

## Discussion

For quantitative discussions of the structure and magnetic properties of the studied sample the PNR data were fitted using the Parratt algorithm in GenX software package^35^. For neutron reflectometry the buffer GaN layer with thickness of 3 *μ*m is considered as the bulk medium. Thus reflectivity data was fitted assuming the model consisted of GaN substrate, transition layer (interface) and *ε*-Fe_2_O_3_ layer. These parameters are summarized in the nuclear scattering length density (SLD) and magnetic SLD profiles (*ρ*_*n*_(*z*) and *ρ*_*m*_(*z*), respectively) shown in Fig. 3b and c. The solid line in Fig. 3b corresponds to the nuclear SLD profile ${\rho }_{n}(z)=\frac{{\sum }_{i\mathrm{=1}}^{N}{b}_{i}}{{V}_{m}}=\frac{\rho {N}_{a}{\sum }_{i\mathrm{=1}}^{N}{b}_{i}}{{\sum }_{i\mathrm{=1}}^{N}{M}_{i}}$ where *b*_*i*_ is scattering length of *i*-th of *N* atoms within the unit cell of volume *V*_*m*_, *ρ* is a density of the material, *M*_*i*_ is the molecular weight, *N*_*a*_ is the Avogadro constant. The initial guess contained the literature values of scattering lengths and densities and nominal thickness of iron oxide layer; then the densities and thicknesses of *ε*-Fe_2_O_3_ and interface layer were allowed to vary freely in the fitting routine. All PNR datasets were fitted simultaneously with the same structural parameters and varying magnetizations of the main and interface layers corresponding to different temperature and field conditions and resolution functions corresponding to the D17 and SHARAKU instruments. The minimum found by the fitting algorithm (goodness of the fit *χ*^2^ = 1.42) corresponds to the model containing interface layer with thickness of *d*_*i*_ = 37 ± 3 Å and roughness *σ*_*i*_ = 24 ± 10 Å between iron oxide and GaN buffer. The obtained density of *ε*-Fe_2_O_3_ layer is reduced by 5% and GaN density is reduced by 1.5% compared to the literature values (dashed lines in Fig. 3b). The deviations from expected bulk values are typical for thin film growth and associated strain, while the larger 5% deviation may be explained by the columnar growth and therefore higher density of defects in the *ε*-Fe_2_O_3_ layer. The nuclear SLD of the interface layer is reduced compared to the main film of *ε*-Fe_2_O_3_ and close to the nominal value of *ρ*_*n*_ of GaFeO_3_ (blue dashed line in Fig. 3b).

Magnetic SLD *ρ*_*m*_ is directly proportional to the magnetization component **M** parallel to **B**: *ρ*_*m*_ = 2.853⋅10^−9^⋅*M* Å^−2^, where *M* is given in emu/cm^3^ units^36^. Therefore, SLD profile *ρ*_*m*_(*z*) represents in-depth distribution of the magnetization of the film. Assuming a homogeneous distribution of magnetization in the sample plane, the magnetization contributions from the main *ε*-Fe_2_O_3_ *m*_*m*_(*B*) and interfacial layers *m*_*i*_(*B*) were correlated through the area-normalized total magnetic moments *m*_*t*_ measured by SQUID: *m*_*t*_⋅(*d*_*m*_ + *d*_*i*_) = *m*_*m*_⋅*d*_*m*_ + *m*_*i*_⋅*d*_*int*_, where *d*_*m*_ and *d*_*i*_ are thicknesses of the main *ε*-Fe_2_O_3_ and interface layers, respectively.

The results of the fitting of magnetic PNR data obtained at 10 K are shown in Fig. 3c,d. The dashed line in Fig. 3c corresponds to the scaled nuclear SLD profile of Fig. 3b for convenience. The solid lines represent the magnetic SLD profiles *ρ*_*m*_(*z*) of at *B* = 2000 mT, 500 mT, 50 mT on the field-decreasing and 100 mT on the field-increasing hysteresis loop branches. The layer-resolved magnetization values measured at *T* = 10 K as a function of magnetic field are summarized in Fig. 3d. Interestingly, the interface layer appears to be magnetically much softer compared to the main *ε*-Fe_2_O_3_ layer. As an illustration, the interface layer remains saturated until relatively small field of 50 mT on the field-decreasing hysteresis loop branch (Fig. 3c) while the magnetization of the main layer is already reduced by half at this point. On the field-increasing branch recorded after applying a negative 2 T field, the main *ε*-Fe_2_O_3_ layer is still in negative remanence at *B* = 100 mT while the interface layer has already switched to positive saturation. This behavior resembles the loops shown in Fig. 2 that demonstrate a pronounced two-step magnetization switching: a wide hysteretic magnetization rotation between −2 T and 2 T, representing the averaged switching of 3 columnar domains, and a narrow zero-remanence magnetization switching with saturation of ≈0.25*M*_*s*_. However, the contribution of the interface layer to the total magnetization is only 0.08 *M*_*s*_ as found from our PNR data. This suggests that another soft magnetic phase is likely present in the sample though cannot be distinguished in the PNR experiment. The most plausible candidates for the soft-magnetic phase are minor *γ*− Fe_2_O_3_ and Fe_3_O_4_ fractions homogeneously distributed within the sample plane^5,20,23^ although not pronounced in X-ray diffraction and X-ray spectroscopy data^15^. In this case further attempts of mapping of the hidden soft-magnetic component in real-space can be performed by means of soft X-ray photoemission electron microscopy. One must also take into account that the columnar structure of the *ε*-Fe_2_O_3_ films observed by TEM suggests that antiphase boundaries are present in considerable concentration between the columns especially in the interface region. As was pointed out in ref.^37^. the antiphase boundaries in iron oxides may account for the soft magnetic behavior. PNR is a laterally averaging technique and only sensitive to long-range ordered moments. Therefore, magnetic moments located in minor phase fractions of small volume, or at the columnar antiphase boundaries cannot be distinguished by this method.

The reduced crystalline order observed by TEM (this work) and RHEED (ref.^15^.) within a few nanometer thick layer at the film - substrate interface is in general agreement with the reduced density. Soft magnetism of the interface region is confirmed by the presented PNR data. The reduced order at the interface however gives no clue as to why the exotic metastable *ε*-Fe_2_O_3_ iron oxide phase nucleates on GaN and moreover grows in the form of epitaxial crystallographically well oriented layers.

A probable explanation can be related to the interfacial gallium sourced in small quantities into the iron oxide film by the GaN substrate heated to 700−800 °C during the growth stage. It is well known that GaN becomes unstable at 900–1000 °C. For example, after 1 hour annealing at *T* = 900 °C in H_2_, thermal degradation of GaN is observed accompanied by formation of individual Ga droplets on the surface^38^. At more moderate conditions an enhanced Ga diffusion is known to occur. Annealing at 900 °C was found to enhance Ga diffusion in Fe_3_O_4_ and Fe_3_O_4_/Ga_2_O_3_ films grown on GaN templates by metal organic chemical vapor deposition (MOCVD), resulting in noticeable mixing at the iron oxide/GaN interface^39,40^.

**Figure 4:**
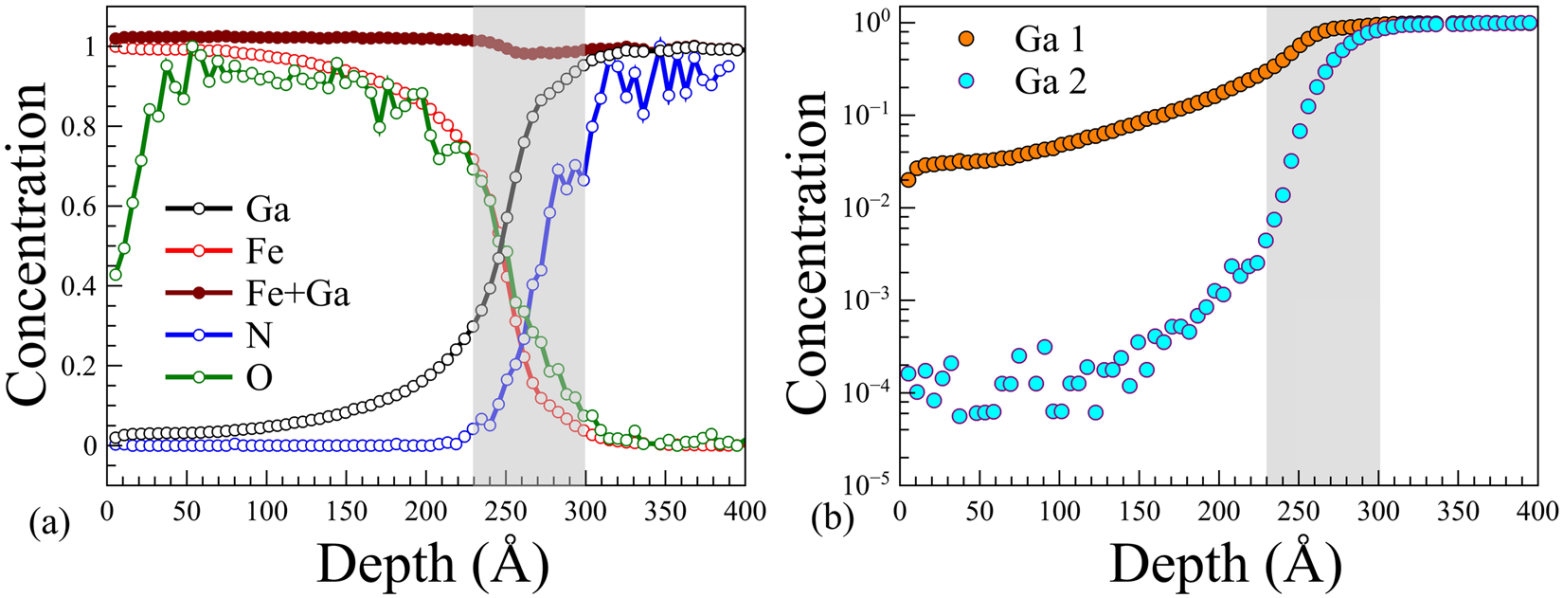
SIMS depth profiles measured in a 25 nm thick *ε*-Fe_2_O_3_/GaN film grown at 800 °C. Iron is presumably substituted by gallium diffusing into the film from the GaN substrate. The gray rectangle gives the idea of broadening related to the film thickness inhomogeneity.

To investigate the chemical composition of *ε*-Fe_2_O_3_/GaN films, SIMS depth profiling was carried out. Sputtering was performed by 1 kV Ar ions (ion current 10 nA) in a 200 *μ*m crater at the rate of 15 s/nm. The mass-spectra of positive secondary ions were collected from a 150 *μ*m area using 25 kV Bi ions. Figure 4 shows the profiles of Fe, O, Ga and N single ions and Ga-Ga pair-ion. One has to take into account that SIMS profiles actually show a convolution of the concentration depth profile with the surface and interface roughness caused by inhomogeneity of the film thickness. This makes it difficult to accurately determine the exact depth corresponding to the Fe_2_O_3_/GaN interface. However using the surface roughness (about 5–7 nm) and the interface roughness (below 2 nm) from AFM, TEM and PNR studies one can readily distinguish two different intensity variation regions in the SIMS profiles. (i) A sharp intensity variation with the width of 5–7 nm present in the profiles of all the monitored elements within the depth region of 230–300 Å is marked with a gray rectangle. We suggest that this variation is due to the surface roughness. (ii) A much slower variation present in the profiles of Ga, Fe and O extending beyond the gray rectangle (depth region of 100–230 Å). It is reasonable to conclude that this variation reflects the actual concentration change. As the Ga concentration is decreasing and Fe is increasing towards the surface, we conclude that the region near the interface is Ga-rich and Fe-deficient. We would like to stress out that the interface region should not be confused with the region marked by the gray rectangle in Fig. 4 as the latter just gives the idea of SIMS profile broadening due to inhomogeneity of the film thickness. Extension of the Ga profile far from the interface into the film region where the profile of N is close to zero suggests diffusion (or any other kind of atomic penetration e.g. floating) of Ga atoms into the film during growth. The effect of Ga atomic penetration is further supported by the Ga-Ga pair-ion profile shown in Fig. 4b. Since the probability of pair-ion formation is proportional to the square of single-ion concentration, one can distinguish concentration variation (pair ion shows twice steeper slope) from inhomogeneity (pair-ion shows the same slope) by comparing single/pair-ion profiles in log scale. Indeed, as can be seen from Fig. 4b the Ga-Ga intensity decay is faster than that of Ga, supporting the idea of atomic penetration. Noteworthy, the Fe intensity is increasing towards the surface almost synchronously with the Ga intensity drop keeping the total intensity of Fe + Ga almost constant across the film. This suggests that gallium substitutes iron in the interface region of the iron oxide layer leading to formation of Ga_*x*_Fe_(1−*x*)_O_3_ compound isostructural to to *ε*-Fe_2_O_3_^41^. Thus one can claim that the interfacial region is Ga rich and Fe deficient as opposed to the top part of the iron oxide film. Formation of gallium ferrite is a probable scenario, which can explain both the magnetic degradation of the Fe_2_O_3_/GaN interface region and the magnetization loop shapes similar to those reported for *ε*-Fe_2_O_3_ layers grown on GaFeO_3_ buffer by Thai *et al*.^8^.

The gallium-assisted scenario of *ε*-Fe_2_O_3_ growth on GaN is the natural assumption at least in view of works where the epsilon-ferrite epitaxial layers were grown on top of a GaFeO_3_ or AlFeO_3_ buffers serving to seed the *Pna*2_1_ crystal structure. In addition, *ε*-ferrite can also be produced in the form of randomly oriented nanocrystals or nanowires by sol-gel technique without any isostructural seeding. Moreover, in a number of works *ε*-Fe_2_O_3_ epitaxial layers were grown without an isostructural buffer^7,9,21^. Importantly, the common feature of all the works on *ε*-Fe_2_O_3_Fe_2_O_3_, to our knowledge, is the nanostructural form of epsilon ferrite–even in epitaxial layers nano-sized columns are observed by means of TEM. Thus one can assume that an important prerequisite for the metastable *ε*-Fe_2_O_3_ phase nucleation is formation of the nanoscale objects. In such objects with high surface-to-volume ratio the energy considerations can favor the formation of a metastable phase otherwise not existing in the bulk form. One of the driving forces that induce granular epitaxial growth is the higher symmetry and smaller lattice constant of the substrate as compared to the iron oxide layer. In this case the grains at the first stage of growth nucleate incoherently upon coalescence thus facilitating columnar growth. Unfortunately, we cannot unambiguously distinguish whether gallium plays the primary role in formation of *Pna*2_1_ GaFeO_3_ and just facilitation of the granular growth of iron oxide on GaN without dedicated experiments. This issue can be further investigated by blocking Ga diffusion through introduction of a barrier layer, such as MgO or Al_2_O_3_ between GaN and *ε*-Fe_2_O_3_. In support of importance of the columnar structure our extended studies of Fe_2_O_3_Fe_2_O_3_ growth on GaN presented in ref.^15^. show that at lower growth temperature (below 800° C) and at lower oxygen pressure (below 0.2 mbar) a more stable *α*-Fe_2_O_3_ phase grows on GaN instead of *ε*-Fe_2_O_3_ as evidenced by *in situ* electron and *ex situ* X-ray diffraction studies. Importantly the *α*-Fe_2_O_3_ layers are considerably more uniform than the *ε*-Fe_2_O_3_ layers. Since the *α*-phase is antiferromagnetic (or weak ferrimagnetic above the Morin temperature), the magnetic properties of the films containing *α*-Fe_2_O_3_ are much deteriorated compared to *ε*-Fe_2_O_3_ films in terms of saturation magnetization and coercive field.

## Conclusion

In the present work a complementary structural, chemical and magnetic in-depth investigation of the *ε*-Fe_2_O_3_ epitaxial thin films grown on GaN (0001) surface by pulsed laser deposition is reported. In agreement with the other works describing the growth of epsilon ferrite films on oxide substrates, the *ε*-Fe_2_O_3_/GaN films were shown to have columnar structure naturally assigned to the presence of the 120° crystallographic domains. Most importantly, a few-nm thick transition layer with distinctly different properties has been detected at the interface between *ε*-Fe_2_O_3_ and GaN. The depth-resolved magnetization profiles obtained by PNR have shown that this interface layer has a lower nuclear SLD and is magnetically softer than the main volume of the *ε*-Fe_2_O_3_ film. While the density variation is in agreement with the transmission electron microscopy measurements, the soft magnetic behavior is shown to be related to the two component magnetization loops observed in the same samples by SQUID magnetometry. Interestingly the SIMS has shown that the interface region is Ga rich and Fe deficient. We conclude that the main *ε*-Fe_2_O_3_ film is likely to inherit the orthorhombic *Pna*2_1_ structure from the isostructural GaFeO_3_-like interface layer, that forms at the early iron oxide deposition stage due to thermal migration of Ga atoms from the substrate. The reported observations shed light onto the mechanisms guiding nucleation of the exotic metastable epsilon ferrite phase on GaN and influencing the structural and magnetic properties of the studied films.

## Electronic supplementary material

Supplementary Information
