# Supplementary material for: Unveiling structural, chemical and magnetic interfacial peculiarities in ε-Fe2O3/GaN (0001) epitaxial films
Source: Sci Rep. 2018 Jun 7;8:8741. doi: 10.1038/s41598-018-25849-z (PMC5992217; doi:10.1038/s41598-018-25849-z)
Supplement: Supplementary file 1 — Supplementary Information [file 41598_2018_25849_MOESM1_ESM.pdf]

Supplemental information for Unveiling structural, chemical and magnetic interfacial peculiarities in ε-Fe_2O_3 / GaN (0001) epitaxial films

Victor Ukleev[1,*,†], Sergey Suturin[2,+], Taro Nakajima[1], Taka-hisa Arima[1,3], Thomas Saerbeck[4], Takayasu Hanashima[5], Alla Sitnikova[2], Demid Kirilenko[2], Nikolai Yakovlev[6], and Nikolai Sokolov[2]

[1]RIKEN Center for Emergent Matter Science (CEMS), Wako 351-0198, Japan
[2]Ioffe Institute, Saint-Petersburg 194021, Russia
[3]Department of Advanced Materials Science, University of Tokyo, Kashiwa 277-8561, Japan
[4]Institut Laue-Langevin, 71 Avenue des Martyrs, 38042 Grenoble, France
[5]Comprehensive Research Organization for Science and Society (CROSS), Tokai, Ibaraki 319-1106, Japan
[6]Institute of Materials Research and Engineering, Agency for Science Technology and Research (A*STAR), 138634 Singapore
[*]victor.ukleev@riken.jp
[†]Current address: Laboratory for Neutron Scattering and Imaging (LNS), Paul Scherrer Institute (PSI), CH-5232 Villigen, Switzerland
[+]suturin@mail.ioffe.ru

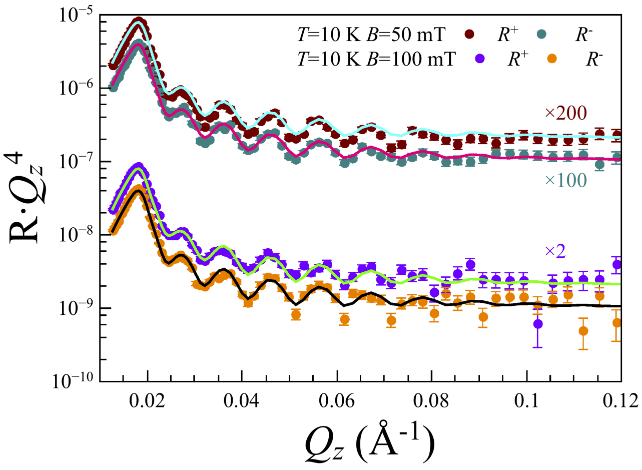

Figure 1. PNR curves measured at $T = 10\,\mathrm{K}$ $B = 50\,\mathrm{mT}$ and $100\,\mathrm{mT}$ upon field reversal. Symbols represents the experimental data while the solid lines are calculated.
